# Ultrasound-Guided Hydrodissection for Neurogenic Thoracic Outlet Syndrome Caused by a Cervical Rib: A Case Report

**DOI:** 10.7759/cureus.104595

**Published:** 2026-03-03

**Authors:** Yonghyun Yoon, Ji Hyo Hwang, Jaeyoung Lee, Jaewoo Lim, Teinny Suryadi, Anwar Suhaimi, Seungbeom Kim, Hyeongjik Kim, King Hei Stanley Lam

**Affiliations:** 1 Orthopedic Surgery, Hallym University Kangnam Sacred Heart Hospital, Seoul, KOR; 2 Orthopedics, IncheonTerminal Orthopedic Surgery Clinic, Incheon, KOR; 3 Orthopedics, International Academy of Regenerative Medicine, Seoul, KOR; 4 Orthopedics, Incheon Terminal Orthopedic Surgery Clinic, Incheon, KOR; 5 Orthopedic Surgery, Incheon Terminal Orthopedic Surgery Clinic, Incheon , KOR; 6 Physical Medicine and Rehabilitation, Synergy Clinic, Jakarta, IDN; 7 Physical Medicine and Rehabilitation, Hermina Podomoro Hospital, Jakarta, IDN; 8 Rehabilitation Medicine, University Malaya Medical Centre, Kuala Lumpur, MYS; 9 Rehabilitation Medicine, University Malaya, Kuala Lumpur, MYS; 10 Pain Medicine, Miso Pain Clinic, Suwon, KOR; 11 Orthopedic Surgery, International Academy of Regenerative Medicine, Seoul, KOR; 12 Faculty of Medicine, The Chinese University of Hong Kong, New Territories, HKG; 13 Faculty of Medicine, The University of Hong Kong, Hong Kong, HKG; 14 The Board of Clinical Research, The Hong Kong Institute of Musculoskeletal Medicine, Kowloon, HKG

**Keywords:** brachial plexus, cervical rib, dextrose injection, dynamic ultrasound, hydrodissection, neurogenic thoracic outlet syndrome

## Abstract

Thoracic outlet syndrome (TOS) is an underdiagnosed condition that is frequently misattributed to cervical spine pathology. Neurogenic TOS (nTOS), the most common subtype, often presents nonspecific upper extremity pain and paresthesia, making diagnosis challenging. We report a case of nTOS associated with a cervical rib that was successfully treated with ultrasound-guided hydrodissection (HD). Dynamic ultrasound examination demonstrated brachial plexus tethering with symptom reproduction. Serial HD resulted in immediate and sustained symptom relief, suggesting that this minimally invasive technique may represent a potential diagnostic adjunct and minimally invasive therapeutic option in carefully selected patients with cervical rib-associated nTOS.

## Introduction

Thoracic outlet syndrome (TOS) encompasses a group of disorders caused by compression of neurovascular structures traversing the thoracic outlet. It is classically classified into neurogenic, arterial, and venous types, with neurogenic TOS (nTOS) accounting for most cases [1]. Patients with nTOS typically present with upper extremity pain, paresthesia, and subjective weakness, often without objective neurologic deficits, leading to frequent misdiagnosis such as cervical disc disease or shoulder pathology [2].

Depending on the anatomical site of compression, TOS can be further subdivided into interscalene, costoclavicular, and pectoralis minor types [3]. Cervical ribs or elongated transverse processes (TPs) may be identified on plain radiographs; however, radiographic findings do not always correlate with symptom severity [4]. Recently, ultrasound has emerged as a useful modality for dynamic assessment of the brachial plexus, allowing real-time evaluation of nerve mobility and symptom reproduction [5]. Dynamic ultrasound offers a unique advantage in neurogenic TOS by allowing real-time assessment of nerve mobility and direct symptom reproduction, which is often not achievable with static imaging modalities [6].

Hydrodissection (HD) is an ultrasound-guided technique that mechanically separates neural structures from surrounding tissues by injecting fluid to create a cleavage plane [7,8]. While HD has been increasingly used for peripheral entrapment neuropathies such as carpal tunnel syndrome, its role in nTOS remains poorly defined. Evidence is particularly limited for HD in nTOS associated with structural anomalies, including a cervical rib. This case highlights the combined diagnostic and therapeutic utility of dynamic ultrasonography and targeted HD in a patient with cervical rib-associated nTOS.

## Case presentation

A 36-year-old female office worker presented with a two-week history of right-sided neck and shoulder pain without preceding trauma. She described sharp, electric shock-like pain radiating along the medial border of the right scapula and extending to the ulnar aspect of the hand, including the fifth digit. Symptoms were aggravated while supporting the arm during walking and were most severe in the morning.

On physical examination, the active and passive shoulder range of motion was full and symmetric. No definite motor weakness was observed, and sensation was intact at rest. Provocative maneuvers, including the Roos and Adson tests, reproduced her symptoms. Cervical radiculopathy was considered unlikely given the absence of dermatomal sensory deficits, preserved deep tendon reflexes, and no symptom exacerbation with the Spurling maneuver. Ulnar neuropathy at the elbow was also considered; however, there was no focal tenderness over the cubital tunnel and no intrinsic hand muscle weakness.

**Figure 1:**
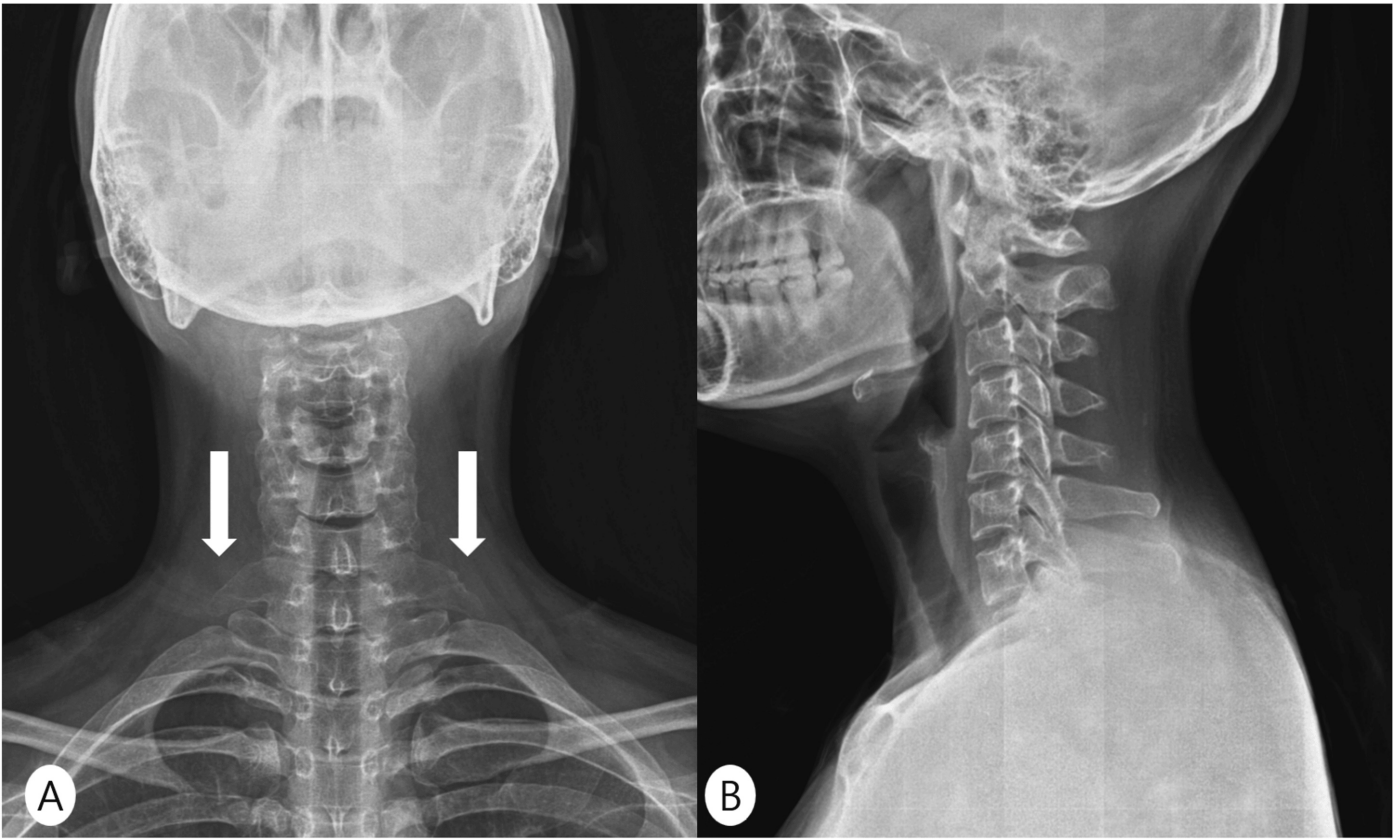
Cervical spine radiographs. (A) Anteroposterior view demonstrating bilateral cervical ribs (arrows). (B) Lateral view showing no remarkable abnormalities.

Plain radiography of the cervical spine demonstrated a bilateral cervical rib on the anteroposterior view (Figure 1A), with no remarkable findings on the lateral view (Figure 1B).

**Figure 2:**
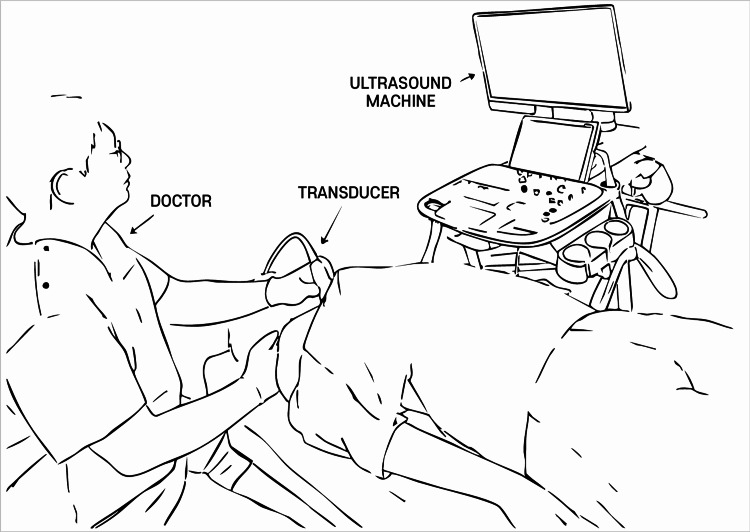
Patient positioning for ultrasound examination and intervention. The patient was placed supine with approximately 15° contralateral neck rotation, and a bolster was positioned under the ipsilateral scapula. Image credit: Created by Yonghyun Yoon using Illustrator

After placing the patient in the supine position, the patient was rotated approximately 15 degrees to the contralateral side, a bolster was placed on the ipsilateral scapula, and the linear probe was examined starting from the first rib and moving upward (Figure 2). This positioning and scanning strategy were adapted from previously described upper-body HD techniques [9].

**Figure 3:**
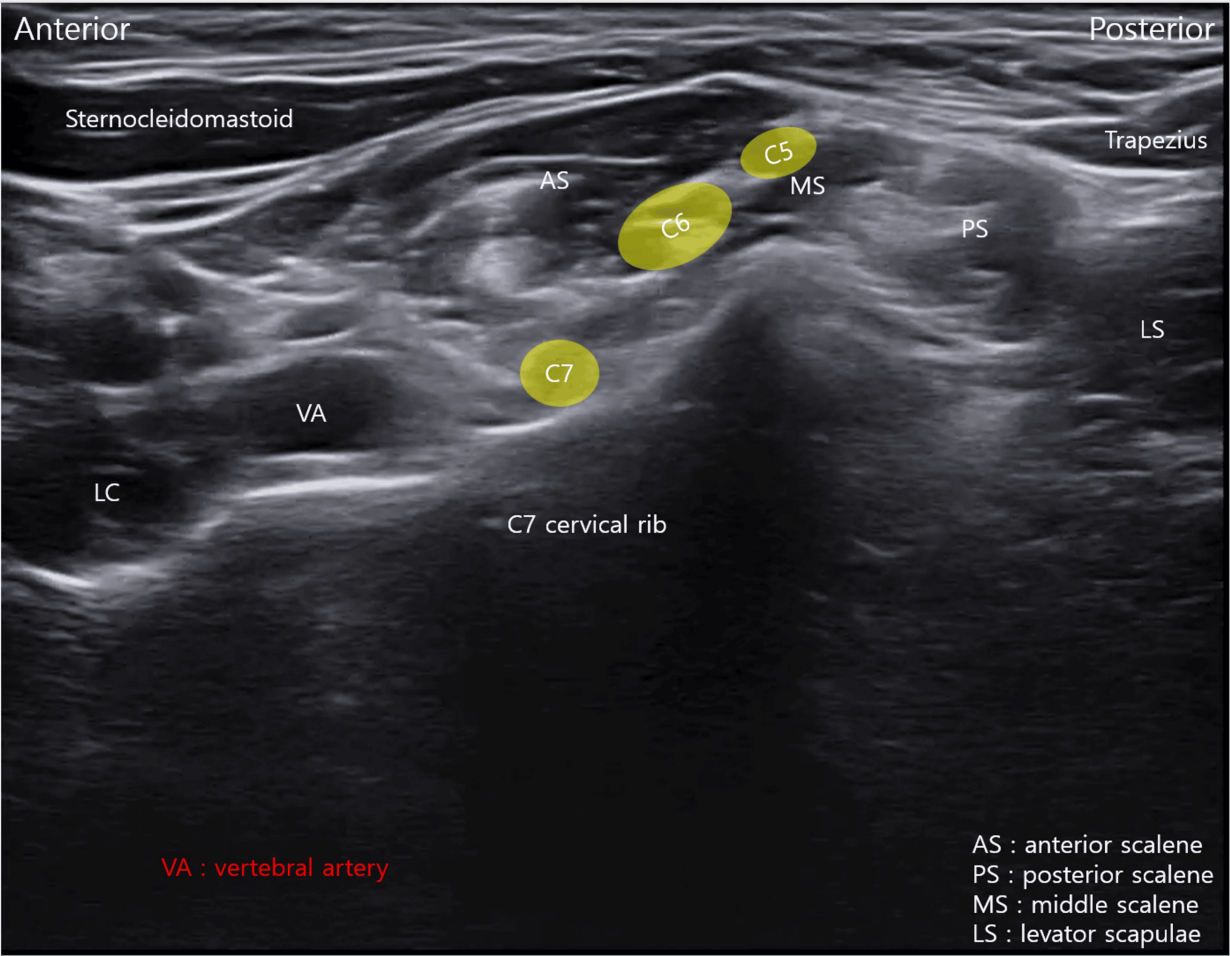
Ultrasound identification of the cervical rib at the C7 level. Long-axis ultrasound view showing an elongated transverse process consistent with a cervical rib on the right side (Video 1).

The C7 TP, located immediately above the first rib, was significantly longer than the other TPs (Figure 3, Video 1).

**Video 1:** Ultrasound evaluation of the cervical rib at the C7 level. The video demonstrates a continuous ultrasound scan from the first rib cranially to the C4 level. An elongated C7 transverse process consistent with a cervical rib is clearly visualized. Key anatomical structures are labeled to facilitate orientation and interpretation.

**Figure 4:**
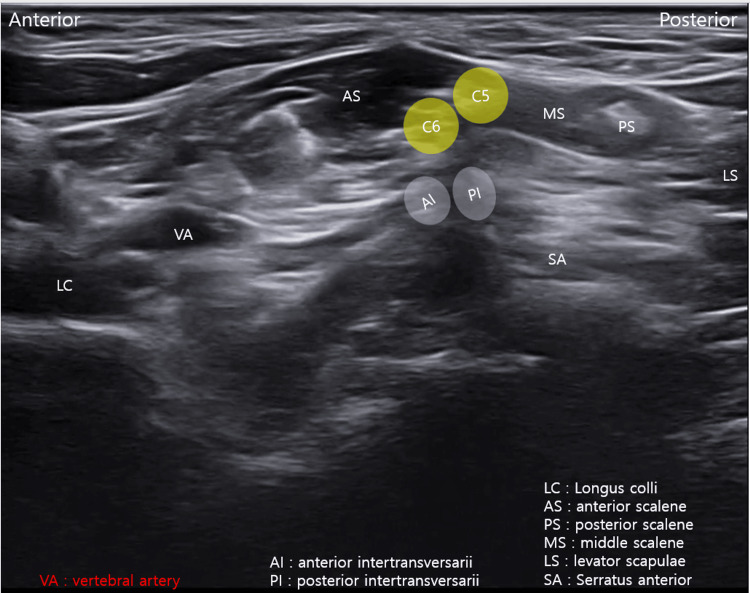
Dynamic ultrasound examination demonstrating brachial plexus tethering with symptom reproduction. With the transducer positioned between the C7 transverse process and the first rib, gentle vertical compression was applied to generate shear across the neurovascular plane, reproducing the patient’s characteristic symptoms (Video 2).

Dynamic ultrasonography was performed with the patient in the same supine position used for the intervention. With the transducer positioned over the interval between the C7 TP and the first rib, graded vertical compression was applied to generate a shear force across the neurovascular plane. During this maneuver, the brachial plexus elements demonstrated approximately 3-4 mm of relative translational displacement with respect to the cervical rib. Direct visualization of brachial plexus tethering with concordant symptom reproduction during dynamic testing strongly supported the diagnosis of nTOS (Figure 4, Video 2).

**Video 2:** Dynamic ultrasound examination for neurogenic thoracic outlet syndrome. With the transducer positioned between the C7 transverse process and the first rib, gentle vertical compression was applied to generate shear across the neurovascular plane. This maneuver demonstrates brachial plexus tethering and reproduces the patient’s characteristic symptoms, supporting the diagnosis of neurogenic thoracic outlet syndrome.

Intervention

**Figure 5:**
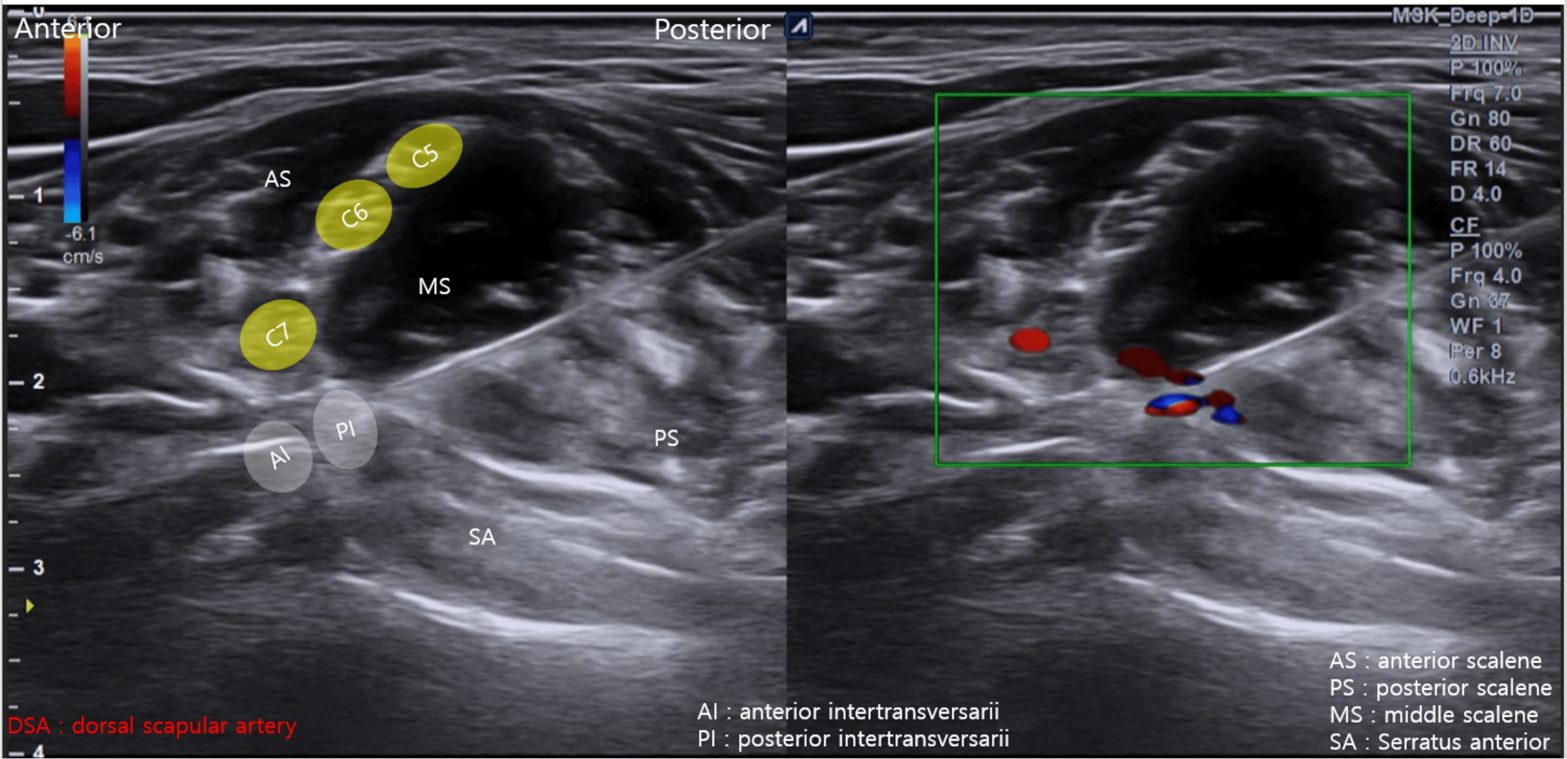
Ultrasound-guided hydrodissection procedure. In-plane posterior-to-anterior approach demonstrating hydrodissection around the brachial plexus, cervical rib, and the C7–T1 intertransversarii muscles (Video 3).

Given the presence of mechanical tethering of the brachial plexus adjacent to the cervical rib, ultrasound-guided HD was selected to release adhesions and reduce neural compression. Ultrasound-guided HD was performed with the patient in a supine position and the neck rotated approximately 15° toward the contralateral side. Using a posterior-to-anterior in-plane approach, HD was carried out around the brachial plexus, cervical rib, and the C7-T1 intertransversarii muscles using 5% dextrose in water (D5W). A total volume of 30 mL was administered (Figure 5, Video 3). The volume of 30 mL was selected to ensure adequate HD across the entire neurovascular plane. The selection of D5W was based on prior clinical evidence demonstrating efficacy in peripheral entrapment neuropathies [10]. Repeated sessions at two-week intervals were performed to allow gradual tissue remodeling and sustained neural mobilization.

**Video 3:** Ultrasound-guided hydrodissection for neurogenic thoracic outlet syndrome. Using a posterior to anterior in-plane approach, hydrodissection is performed around the brachial plexus and the C7–T1 intertransversarii muscles with 5% dextrose in water. The procedure demonstrates mechanical separation of the neural structures from surrounding tissues to reduce neural compression.

Because frequent in-person visits were not feasible, the patient was instructed about a home exercise program focusing on cervical extension and range-of-motion exercises and attended physical therapy twice weekly. Ultrasound-guided HD was performed and repeated at two-week intervals for a total of five sessions.

Results

After the first ultrasound-guided HD session, the patient reported immediate improvement in paresthesia, with the visual analog scale (VAS) score decreasing from 9 to 3. HD was repeated at two-week intervals for a total of five sessions. Following the final session, the patient reported complete resolution of pain and paresthesia (VAS 0).

Functional status also improved, with the Quick Disabilities of the Arm, Shoulder and Hand (QuickDASH) score decreasing from 21 to 2 (Table 1). At the three-month follow-up, she remained symptom-free with no recurrence.

**Table 1:** Summary of clinical features, diagnostic findings, treatment protocol, and outcomes. This table summarizes the patient’s baseline clinical presentation, key diagnostic findings including dynamic ultrasonography, treatment protocol details, and objective outcome measures following ultrasound-guided hydrodissection.

Variable	Finding
Age/Sex	36-year-old female
Symptom duration	2 weeks
Key symptoms	Ulnar-side radiating pain
Provocative tests	Roos (+), Adson (+)
Dynamic US finding	3–4 mm translational displacement
Treatment	HD, 30 mL D5W, 5 sessions
VAS	9 → 0
QuickDASH	21 → 2
Follow-up	No recurrence at three months

## Discussion

nTOS associated with a cervical rib poses substantial diagnostic and therapeutic challenges. To our knowledge, limited reports have described the combined use of dynamic ultrasonography to reproduce symptoms and ultrasound-guided targeted HD in cervical rib-associated nTOS [11]. In this case, the coexistence of a structural anomaly and real-time functional confirmation on dynamic ultrasound distinguishes our approach from prior reports of HD performed for more distal entrapment neuropathies.

Conservative management remains the first-line treatment for nTOS and typically includes activity modification, postural correction, physical therapy focusing on scapular stabilization, and pharmacologic pain control [1]. Many patients improve with structured rehabilitation, particularly in the absence of objective neurologic deficits. Surgical decompression, including cervical rib resection or first rib resection with scalenectomy, may be considered in refractory cases or when progressive neurologic impairment is present. Although surgery can provide substantial symptom relief in appropriately selected patients, it carries procedural risks and variable outcomes [12]. In the present case, given the limited symptom duration, absence of motor deficit, and lack of progressive neurologic findings, a conservative approach incorporating targeted HD was pursued prior to considering surgical intervention. Conventional therapy alone, however, may be insufficient when mechanical tethering of the brachial plexus serves as the primary pain generator.

This case highlights several clinically important points. First, dynamic ultrasound examination enabled direct visualization of brachial plexus tethering and reproduced the patient’s characteristic symptoms in real time, reinforcing its value as a diagnostic adjunct in nTOS. Unlike static imaging modalities, dynamic ultrasound permits functional assessment of nerve mobility, which can be critical in anatomically complex cases [13].

Second, ultrasound-guided HD resulted in both immediate and sustained symptom relief. By mechanically separating the brachial plexus from surrounding structures, including the cervical rib and adjacent musculature, HD may reduce neural compression and irritation. This observation aligns with prior evidence supporting the use of HD in peripheral entrapment neuropathies. In carpal tunnel syndrome, ultrasound-guided HD has been shown to improve symptoms and functional outcomes by restoring median nerve mobility and reducing intraneural edema [10]. Similar benefits have been reported in other entrapment conditions, including ulnar neuropathy at the elbow and meralgia paresthetica, where mechanical separation of the nerve from surrounding fibrotic or compressive tissues resulted in meaningful clinical improvement. When a clear anatomical contributor to compression is identified, HD may therefore represent a minimally invasive therapeutic option by facilitating neural gliding and decreasing mechanical irritation. In patients with cervical rib-associated nTOS, targeted HD may be considered a rational extension of this established principle [14,15]. Recent clinical evidence has also begun to emerge in nTOS, including a 2025 retrospective application study reporting improvement in pain and functional outcomes following ultrasound-guided HD-based intervention for neurogenic thoracic outlet syndrome [11].

D5W was selected based on emerging evidence suggesting both mechanical and potential neuromodulatory effects [10,16]. In addition to facilitating physical separation of adhesions, dextrose has been proposed to reduce neurogenic inflammation and ectopic neural discharge. The injectate volume of 30 mL was chosen to ensure adequate mechanical separation across the costoclavicular neurovascular plane, which is anatomically broader than distal entrapment sites [8]. In carpal tunnel syndrome, a prospective randomized study demonstrated that injectate volume may influence clinical and ultrasonographic outcomes, supporting the principle that sufficient fluid is required for effective perineural separation [17]. Case reports involving proximal radial nerve-related entrapment neuropathies have similarly described the use of larger injectate volumes (approximately 30-50 mL) to achieve adequate tissue-plane separation and neural gliding [12,18]. Although anatomical contexts differ, these findings suggest that broader neurovascular planes may require higher injectate volumes to restore neural mobility. Serial sessions at two-week intervals were performed to facilitate progressive restoration of perineural gliding and sustained symptom relief, consistent with repeated-injection protocols reported in peripheral nerve HD studies [10].

This report is limited by its single-patient design. The long-term durability of symptom relief following HD remains uncertain, and the absence of a control group precludes exclusion of nonspecific effects such as placebo response or natural symptom fluctuation. Further studies are warranted to establish standardized protocols, define patient selection criteria, and evaluate long-term outcomes of ultrasound-guided HD in nTOS.

## Conclusions

Ultrasound-guided HD may represent a potential adjunctive option for carefully selected patients with cervical rib-associated nTOS. In this case, dynamic ultrasound assessment combined with targeted HD was associated with meaningful and sustained symptom improvement. Larger studies are warranted to clarify patient selection criteria and to establish optimal treatment protocols.

## References

[1] Illig KA, Donahue D, Duncan A (2016). Reporting standards of the Society for Vascular Surgery for thoracic outlet syndrome. J Vasc Surg.

[2] Lim C, Kavousi Y, Lum YW, Christo PJ (2021). Evaluation and management of neurogenic thoracic outlet syndrome with an overview of surgical approaches: a comprehensive review. J Pain Res.

[3] Ahmed AS, Lafosse T, Graf AR, Karzon AL, Gottschalk MB, Wagner ER (2023). Modern treatment of neurogenic thoracic outlet syndrome: pathoanatomy, diagnosis, and arthroscopic surgical technique. J Hand Surg Glob Online.

[4] Demondion X, Herbinet P, Van Sint Jan S, Boutry N, Chantelot C, Cotten A (2006). Imaging assessment of thoracic outlet syndrome. Radiographics.

[5] Fried SM, Nazarian LN (2013). Dynamic neuromusculoskeletal ultrasound documentation of brachial plexus/thoracic outlet compression during elevated arm stress testing. Hand (N Y).

[6] Khalilzadeh O, Glover M, Torriani M, Gupta R (2021). Imaging assessment of thoracic outlet syndrome. Thorac Surg Clin.

[7] Lee K, Park JM, Yoon SY, Kim MS, Kim YW, Shin JI, Lee SC (2025). Ultrasound-guided nerve hydrodissection for the management of carpal tunnel syndrome: a systematic review and network meta-analysis. Yonsei Med J.

[8] Lam KH, Hung CY, Chiang YP, Onishi K, Su DC, Clark TB, Reeves KD (2020). Ultrasound-guided nerve hydrodissection for pain management: rationale, methods, current literature, and theoretical mechanisms. J Pain Res.

[9] Lam SK, Reeves KD, Cheng AL (2017). Transition from deep regional blocks toward deep nerve hydrodissection in the upper body and torso: method description and results from a retrospective chart review of the analgesic effect of 5% dextrose water as the primary hydrodissection injectate to enhance safety. Biomed Res Int.

[10] Wu YT, Ho TY, Chou YC, Ke MJ, Li TY, Tsai CK, Chen LC (2017). Six-month efficacy of perineural dextrose for carpal tunnel syndrome: a prospective, randomized, double-blind, controlled trial. Mayo Clin Proc.

[11] Liao Z, Zhou Y, Cao W (2025). Ultrasound-guided the prevertebral fascia incise and the C5 root hydrodissection for the treatment of neurogenic thoracic outlet syndrome: an application study. J Pain Res.

[12] Peek J, Vos CG, Ünlü Ç, van de Pavoordt HD, van den Akker PJ, de Vries JP (2017). Outcome of surgical treatment for thoracic outlet syndrome: systematic review and meta-analysis. Ann Vasc Surg.

[13] Yoon Y, Lam KH, Castro JC (2026). Ultrasound-guided dextrose hydrodissection for mixed sensory-motor Wartenberg's syndrome following a healed scaphoid fracture: a case report. Diagnostics (Basel).

[14] Tranchitella NM, Pottanat PJ, Sherrier M (2024). Ulnar nerve hydrodissection at the elbow with ultrasound guidance. Video J Sports Med.

[15] Shi X, Xu H, Zhu J, Li G, Bai L (2024). A randomized double-blind trial of 5% dextrose versus corticosteroid hydrodissection for meralgia paresthetica. Pain Physician.

[16] Lyftogt J (2007). Subcutaneous prolotherapy treatment of refractory knee, shoulder, and lateral elbow pain. Australasian Musculoskeletal Med.

[17] Huang CY, Lai CY, Reeves KD, Lam KH, Li TY, Cheng CI, Wu YT (2024). Volume effect of nerve hydrodissection for carpal tunnel syndrome: a prospective, randomized, and single-blind study. J Ultrasound Med.

[18] Yoon Y, Lam KH, Lee J (2025). An ultrasound image-and-intervention paradigm for a neglected lumbar transverse process fracture: a case report of diagnosis and dydrodissection treatment. Cureus.

